# MetNet: Software to Build and Model the Biogenetic Lattice of *Arabidopsis*

**DOI:** 10.1002/cfg.285

**Published:** 2003-04

**Authors:** Eve Syrkin Wurtele, Jie Li, Lixia Diao, Hailong Zhang, Carol M. Foster, Beth Fatland, Julie Dickerson, Andrew Brown, Zach Cox, Dianne Cook, Eun-Kyung Lee, Heike Hofmann

**Affiliations:** 1 Department of Genetics Cellular and Developmental Biology Iowa State University Ames IA 50011 USA; 2 Bioinformatics and Computational Biology Program Iowa State University Ames IA 50011 USA; 3 Department of Statistics Iowa State University Ames IA 50011 USA; 4 Department of Electrical and Computer Engineering Iowa State University Ames IA 50011 USA

## Abstract

MetNet (http://www.botany.iastate.edu/∼mash/metnetex/metabolicnetex.html) is publicly available software in development for analysis of genome-wide RNA, protein and metabolite profiling data. The software is designed to enable the biologist to visualize, statistically analyse and model a metabolic and regulatory network map of *Arabidopsis*, combined with gene expression profiling data. It contains a JAVA interface to an interactions database (MetNetDB) containing information on regulatory and metabolic interactions derived from a combination of web databases (TAIR, KEGG, BRENDA) and input from biologists in their area of expertise. FCModeler captures input from MetNetDB in a graphical form. Sub-networks can be identified and interpreted using simple fuzzy cognitive maps. FCModeler is intended to develop and evaluate hypotheses, and provide a modelling framework for assessing the large amounts of data captured by high-throughput gene expression experiments. FCModeler and MetNetDB are currently being extended to three-dimensional virtual reality display. The MetNet map, together with gene expression data, can be viewed using multivariate graphics tools in GGobi linked with the data analytic tools in R. Users can highlight different parts of the metabolic network and see the relevant expression data highlighted in other data plots. Multi-dimensional expression data can be rotated through different dimensions. Statistical analysis can be computed alongside the visual. MetNet is designed to provide a framework for the formulation of testable hypotheses regarding the function of specific genes, and in the long term provide the basis for identification of metabolic and regulatory networks that control plant composition and development.


**Comparative and Functional Genomics**
*Comp Funct Genom* 2003; **4**: 239–245.
Published online 1 April 2003 in Wiley InterScience (www.interscience.wiley.com). **DOI:** 10.1002/cfg.285

**Conference Review**

### MetNet: software to build and model the biogenetic lattice of *Arabidopsis*

Eve Syrkin Wurtele[1]*, Jie Li[1,2], Lixia Diao[1,3], Hailong Zhang[2], Carol M. Foster[1], Beth Fatland[1], Julie Dickerson[2,4], Andrew Brown[4], Zach Cox[4], Dianne Cook[3], Eun-Kyung Lee[3] and Heike Hofmann[3]
[1]*Department of Genetics, Cellular and Developmental Biology, Iowa State University, Ames IA 50011, USA*
[2]*Bioinformatics and Computational Biology Program, Iowa State University, Ames IA 50011, USA*
[3]*Department of Statistics, Iowa State University, Ames IA 50011, USA*
[4]*Department of Electrical and Computer Engineering, Iowa State University, Ames IA 50011, USA*

**Correspondence to:*
*Eve Syrkin Wurtele, Iowa State University, Ames IA 50011, USA.*
*E-mail: mash@iastate.edu*

Received: 4 February 2003
Revised: 7 February 2003
Accepted: 10 February 2003

### Abstract

**MetNet (http://www.botany.iastate.edu/~mash/metnetex/metabolicnetex.html) is publicly available software in development for analysis of genome-wide RNA, protein and metabolite profiling data. The software is designed to enable the biologist to visualize, statistically analyse and model a metabolic and regulatory network map of *Arabidopsis*, combined with gene expression profiling data. It contains a JAVA interface to an interactions database (MetNetDB) containing information on regulatory and metabolic interactions derived from a combination of web databases (TAIR, KEGG, BRENDA) and input from biologists in their area of expertise. FCModeler captures input from MetNetDB in a graphical form. Sub-networks can be identified and interpreted using simple fuzzy cognitive maps. FCModeler is intended to develop and evaluate hypotheses, and provide a modelling framework for assessing the large amounts of data captured by high-throughput gene expression experiments. FCModeler and MetNetDB are currently being extended to three-dimensional virtual reality display. The MetNet map, together with gene expression data, can be viewed using multivariate graphics tools in GGobi linked with the data analytic tools in R. Users can highlight different parts of the metabolic network and see the relevant expression data highlighted in other data plots. Multi-dimensional expression data can be rotated through different dimensions. Statistical analysis can be computed alongside the visual. MetNet is designed to provide a framework for the formulation of testable hypotheses regarding the function of specific genes, and in the long term provide the basis for identification of metabolic and regulatory networks that control plant composition and development. Copyright © 2003 John Wiley & Sons, Ltd.**

**Keywords:** ***Arabidopsis*; metabolic network; regulatory network; fuzzy cognitive map; gene expression; metabolomics**

### Introduction

A major challenge in the post-genome era is to understand how interactions among molecules in a cell determine its form and function. With the help of transcriptomic, proteomic and metabolomic analysis technologies, biologists can obtain vast amounts of valuable data on gene expression on a global scale, and many approaches are being developed to analyse the resultant data [e.g. 1,5,7,12,15,17]. Several of these approaches use complex databases of cellular interactions. The WIT Project [13] (**http://wit.mcs.anl.gov/WIT2/WIT**) and the Kyoto Encyclopedia of Genes and Genomes [9] (KEGG) (**http://www.genome.ad.jp/kegg**) provide molecular networks based on prokaryotic metabolism. WIT produces reconstructions of the metabolism of the organism derived from sequence, biochemical and phenotypic data, organized

Copyright © 2003 John Wiley & Sons, Ltd.

as a static presentation. KEGG's goals are to computerize existing knowledge of the information pathways that consist of interacting genes or molecules and to link individual components of the pathways with the gene catalogues being produced by the genome projects. This approach provides the framework for eventual simulations. E-CELL is a set of software tools that enables a user to model cellular interactions to conduct experiments '*in silico*', specifying the molecules, the interactions between these molecules, and then numerically integrating the differential equations described implicitly in these reaction rules to compute how they work together as a system [15]. Using data from the WIT database, E-CELL has been used to construct a hypothetical cell containing 127 genes.

EcoCyc is a pathway/genome database for *Escherichia coli* that describes its enzymes, and its transport proteins (**http://www.ecocyc.org/**). MetaCyc describes pathways and enzymes for many different organisms [10]. The databases combine information from a number of sources and provide function-based retrieval of DNA or protein sequences. EcoCyc has made significant advances in visualizing metabolic pathways using stored layouts, linking data from microarray tests to the pathway layout, and ontology development [11].

MetNet is designed to provide a framework for the formulation of testable hypotheses regarding the function of specific genes, proteins and metabolites and, in the long term, provide the basis for identification of genetic regulatory networks that control plant composition and development [2–6,12]. Our focus is on the eukaryotic model plant, *Arabidopsis*. The entire *Arabidopsis* genome has been sequenced, and databases cataloguing genes and gene function, as well as publicly available collections of insertional mutations in individual genes, are expanding. New databases, e.g. of protein–protein interactions, are being initiated. However, more than half of the 26 000 *Arabidopsis* genes have no assigned function, and many of the remaining genes have only putatively-assigned biochemical functions. Even less is understood about the metabolic, structural or regulatory role of each gene product, its interactions with other cellular components and the kinetics of each interaction. Our approach to reveal complex biological networks is to extract information from gene expression data sets and combine it with what we already know about metabolic and regulatory pathways. The tools within MetNet include capabilities for: (a) mapping metabolic and regulatory networks, including descriptions of subcellular compartmentation of the entities and the characteristics of the interactions between them; (b) integrating and visualizing data together with statistical and clustering packages; (c) exploring and modelling the metabolic and regulatory flow in the network. Herein, we describe the form of the metabolic and regulatory map of *Arabidopsis*, and give examples of how the map can be integrated with our clustering, visualization and modelling tools.

### Results

### Creating a metabolic and regulatory map

The Metabolic Networking Data Base (MetNetDB) will contain a metabolic and regulatory map of *Arabidopsis*, with a user-friendly JAVA interface for creating and searching the map. The map is continuous; this absence of discrete pathways is important for modelling and also for visualizing the variety of networks and pathways in the map. The map, together with gene expression data (metabolomics, proteomics and microarray), can be transferred to FCModeler and GGobi as an XML file, for use in data exploration (see also Figures 1 and 2).

The MetNetDB map is being assembled by biologists with expertise in specific areas of metabolism. It is composed of entities (genes, RNAs, polypeptides, protein complexes, metabolites and environmental inputs) connected by interactions (conversion, catalytic, regulatory). The identities of the genes, RNAs, and polypeptides have been downloaded from TAIR (**http://www.arabidopsis.org/**). Protein complexes are currently added by expert users, as there is no adequate database of protein complexes in *Arabidopsis*. The identities of many metabolites have been downloaded from KEGG (**http://www.genome.ad.jp/kegg/**); metabolites not present in KEGG are manually added as new entities by expert users, based on their CAS registry number (**http://www.cas.org/EO/regsys.html**). The metabolic reactions from Aracyc (**http://www.arabidopsis.org/tools/aracyc/**) have been downloaded into MetNetDB. Expert users can draw from these reactions; the associated entities must be assigned subcellular

Copyright © 2003 John Wiley & Sons, Ltd.

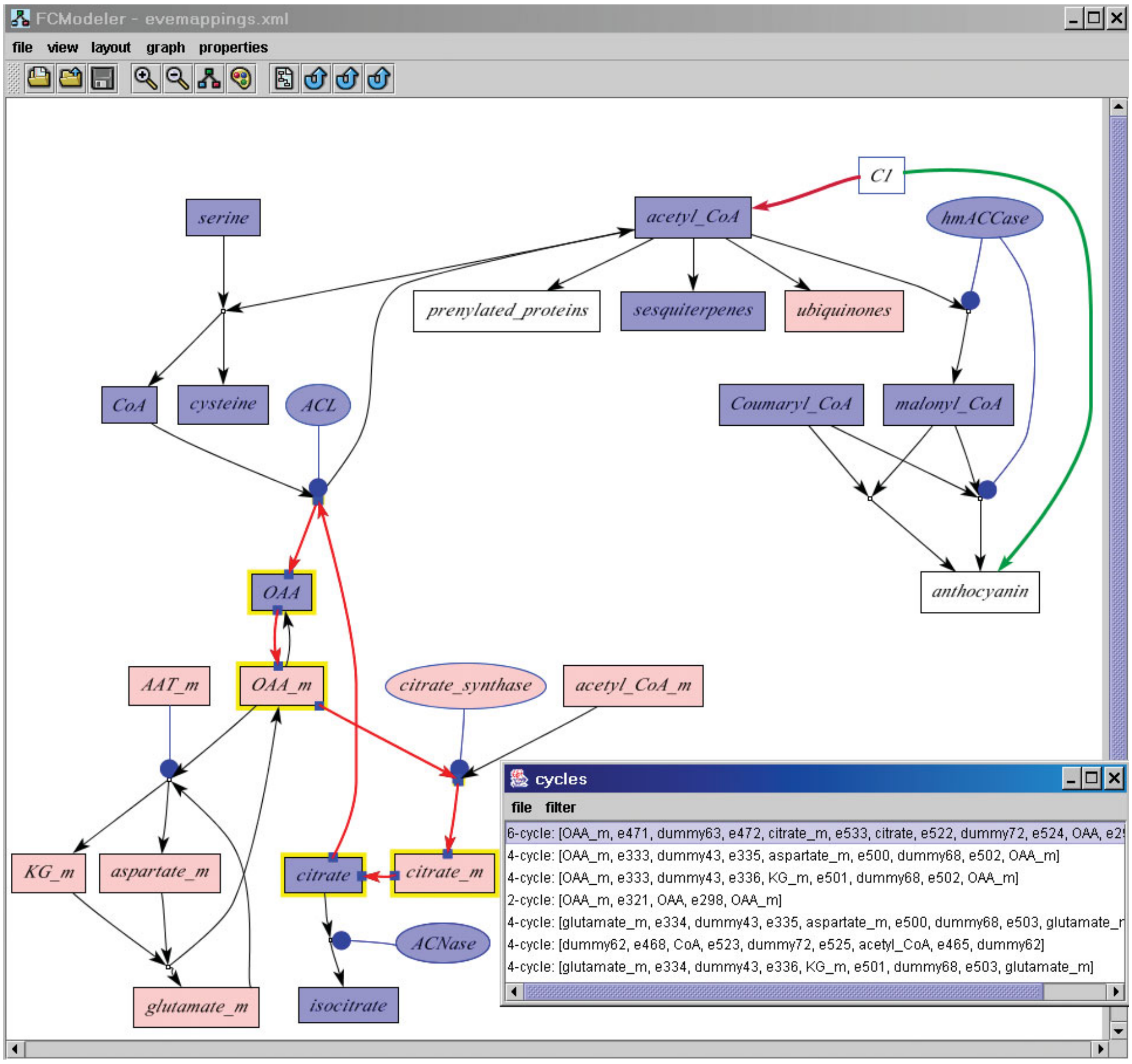

**Figure 1.** The highlighted nodes and links show a small cycle within the metabolic network. The textbox gives a list of all cycles found in the displayed graph. Colours and shapes of features can be user-designated. Entities in mitochondria are: pink, entities in cytosol; blue, entities in unknown location; white, enzymes are shaped as ellipses locations before entry into the map. Interactions not yet present in MetNetDB are created by the expert users. An important aspect of the map is the inclusion of information on subcellular location. This is critical, because particular entities can interact, contingent on being located in the same subcellular compartment. A given entity may be present as separate pools in multiple compartments, e.g. citrate is present in the mitochondria (where it participates in the TCA cycle) and the cytosol (where it is a substrate for cytosolic acetyl-CoA formation [8]).

Three basic types of interactions are specified. In a *conversion* interaction, a node, typically a chemical(s), is converted into another node and used up in the process. A *catalytic* interaction represents an enzyme that enables a chemical conversion and does not get used up in the process. In a *regulatory* interaction, the entity activates or deactivates another node and is not used up in the process. A wide variety of cellular processes can be represented, each occurring to entities in specified subcellular compartments, e.g. to represent the reaction catalysed by ATP

Copyright © 2003 John Wiley & Sons, Ltd. 

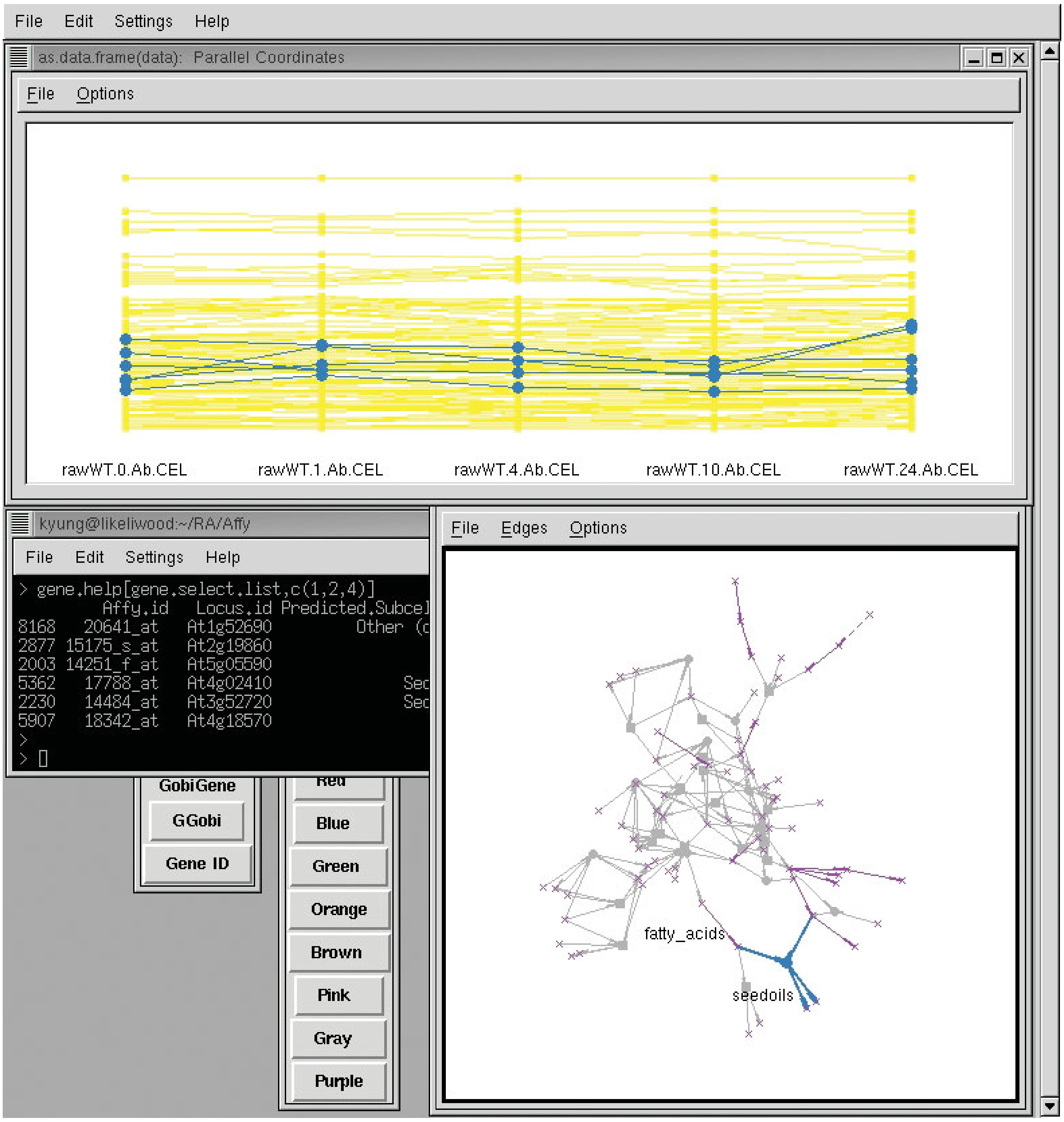

**Figure 2.** Screen shot of GeneGobi analytic tools. At the top is a view of gene profiles over time, with genes of interest selected (large solid circles, darker lines). In the middle is the network view with the relevant part of the network highlighted, alongside a text window from R containing information about the selected genes. Two control panels for statistical selection are at bottom left citrate lyase (ACL) that generates cytosolic acetyl-CoA [13], two interactions are used. One is a *conversion* interaction; its inputs are citrate$_{cytosol}$ + CoA$_{cytosol}$ + ATP$_{cytosol}$ and its outputs are acetyl-CoA$_{cytosol}$ + oxaloacetic acid$_{cytosol}$ + ADP$_{cytosol}$ + P0$_{4cytosol}$. The second is a *catalytic* interaction; its input is ATP citrate lyase$_{cytosol}$. In another example, to represent the translocation of citrate from the mitochondrion to the cytosol, two entities and a single *conversion* interaction are used: citrate$_{mitochondrion}$ goes to citrate$_{cytosol}$. The formation or modification of a protein complex can also be represented, e.g. ACLA and ACLB are the subunits that compose the enzyme ACL. A single *conversion* interaction is used to represent the reaction; its inputs are $ACLA_{cytosol}$, and $ACLB_{cytosol}$. Its output is $ACL_{cytosol}$. The accumulation of the gibberellin-induced RNA GASA in the presence of the plant hormone gibberellin is represented as a *regulatory* interaction; GA4 is the input and GASA-RNA is the output. This is an example of a regulatory interaction for which many details are currently missing.

Because our current understanding of cellular interactions is incomplete, biological systems can be modelled only in approximation. As more experimental data and new databases become available, the MetNetDB is designed to incorporate this new information. Kinetic constants for interactions can be added to MetNetDB as data becomes available; this information can be incorporated into the modelling of the data. Thus, a key consideration in designing MetNetDB is to provide an efficient way for the expert user to determine whether corrections or additions are needed and to input those changes seamlessly.

### Graphing, evaluating and modelling the metabolic and regulatory map

The main goals of the FCModeler package are to capture the intuitions of biologists and provide a modelling framework for assessing large amounts of information and to test the effects of hypotheses. The tools that are being developed use graph theoretic approaches to analyse network structure and behaviour and fuzzy methods that model changes in the network [5]. There are three parts of this system: a dynamic graph visualization package written in Java; network analysis to find critical paths; and modelling using fuzzy cognitive maps to capture uncertainty in the model. Figure 1 shows a sample sub-graph from the MetNetDB for OAA metabolism in *Arabidopsis* and highlights some of the visualization flexibility of the program.

The interactions (also referred to as edges or links) in the network are modelled as fuzzy functions, depending on the detail known about the network. Modelling using fuzzy cognitive maps (FCMs) is performed in the Matlab™ analysis program and the results showing node activation levels are animated in FCModeler. Fuzzy cognitive maps are fuzzy digraphs that model causal flow between concepts [14] or, in this case, biomolecular entities [3,5]. Entities stand for causal fuzzy sets where events occur to some degree. The entities are linked by interactions that show the degree to which these entities depend on each other. Interactions stand for causal flow. The sign of an interaction (+ or −) shows causal increase or decrease between entities. The fuzzy structure allows the RNA, metabolite or protein levels to be expressed as continuous values.

Complex metabolic networks can be analysed by searching for paths between two entities and assessing the effects that the entities have on each other or by searching for feedback cycles in the network as shown in Figure 1. These cycles often show regulation in the network, such as for gibberellin conversion from an inactive form to an active form [3]. Preliminary results have found existing pathways in the map as well as new relationships in the models. Another analysis tool is the diffusion algorithm which assesses how strongly one entity influences another. The output graph shows the strength of the interaction implicit in the network model.

Future FCModeler development plans include the incorporation of different levels of information into the fuzzy cognitive maps, such as functional interactions and kinetic values, finding critical points in interacting pathways, and the visualization of graphs in 3D virtual reality systems to help understand the effects of physical location within the cell on the model.

### Visual and analytic tools

For the analysis of global gene expression experiments (microarray, proteomics or metabolomics), special tools are required. Even though each experiment may be done in a different setting with a different question in mind, many of these experiments have common goals and a similar design. Quite often, gene expression is measured at several points in time or over space, and in multiple genotypes. Questions about the data can be classified into several types; all of them involve the identification of a small number of 'interesting' genes within a single experiment, or across multiple experiments:

- *'Compared to' questions*, e.g. which genes are overexpressed in the genetically modified plants relative to the wild-type (at a given time point)?

Copyright © 2003 John Wiley & Sons, Ltd.

- *Trends or profile matches*, e.g. which genes are upregulated over the time course? Which genes have a peak of expression at the first time point and then flatten out?
- *Comparisons of profiles*, e.g. which genes have different profiles over time in the modified genotype compared to the wild-type?
- *Identification of distinguishing patterns of gene expression*, e.g. define a gene profile that characterizes this genotype relative to other genotypes.

To answer these questions, analytical and visual tools need to be adjusted to the special requirements of the data. The advantage of the interactive environment here, using graphics together with statistical computation, is that selections and combinations of different measures are flexible. We are using statistical analyses from R (**http://www.r-project.org**) in combination with our visual tools. One statistical issue is normalizing values across experiments to allow them to be compared. The BioConductor Project (**http://www.bioconductor.org**) is a package focusing on microarray data that is being developed as an extension to R, e.g. BioConductor provides methods for reading files and normalization of the gene expression data. In combination with R, powerful statistical analysis methods are then available for use on the normalized data.

R can be used for tasks such as defining simple statistical measures to express similarities/dissimilarities, e.g. the correlation coefficient provides a measure for the amount of linear relationship among objects: the correlation between time and expression levels of a gene hints at the amount of upwards or downwards regulation of the gene over time. A high positive correlation between the expression levels of two genes indicates a similar regulation pattern. Another measure for similarity is the Euclidean distance. A small distance in expression levels of two genes is in indicator for very similar expression levels. These distance measures can be computed quickly and built into functions that will generate lists of genes that behave in certain ways. R also has tools to bring up a web browser to search for more information in the public databases for literature about the genes.

Exploration of expression data begs for graphics that allow the user to directly query, modify and change plot elements. This greatly enhances the biologist's ability to answer the questions. Graphical methods have the advantage of being user friendly, intuitive and very flexible. The analysis is driven by the data. Graphical displays that plot expression values in different views, as histograms, scatterplots or profiles over time, and allow the analyst to query genes, mark or highlight groups of genes, make comparisons between genotypes simpler. Implementations of these ideas can be found in the software MANET [16] (**http://www.rosuda.org/Manet**) and GGobi [14] (**http://www.ggobi.org**). GGobi is linked tightly to R, making communication between these two softwares easy. Figure 2 shows a screen shot of this interaction. At the top is a parallel coordinate plot showing profiles of gene expression levels over time. Highlighted in blue are five genes that have been selected by a statistical determination as being interesting. The listing in the middle gives a short summary of these genes, including the genes' Affymetrix ID and locus. A separate window, containing the gene annotations and links to TAIR, SWISSPROT and GO, can also be queried. The menu windows at bottom left belong to the graphical user interface to the R functions used for querying the expression levels. At the bottom right of Figure 2 the corresponding metabolic network is laid out. The highlighted blue part corresponds to the blue marked genes of the upper diagram. Many gaps remain in our understanding of the interactions in the metabolic and regulatory network map. Using current knowledge about how genes are a part of subgraphs within the network, we will examine the expression data to find similarly behaving genes and to piece together the puzzle of genetic control of metabolic function.

### Summary

We have designed software with a focus on understanding the complex molecular network in the model plant, *Arabidopsis*. Our software tools enable biologists to capture relationships at different levels of detail, to integrate gene expression data and to model these relationships. Because of our absence of knowledge about many biological interactions, the software is designed to model at many levels of detail. A future aim is to use gene expression data to

Copyright © 2003 John Wiley & Sons, Ltd. 

suggest new causal interactions in *Arabidopsis* biology.

### Acknowledgements

We thank Lucas Mueller and TAIR for helpful advice and Aracyc data. This work is supported by grants from NSF (MCB-9998292 and *Arabidopsis* 2010 DBI-0209809), USDA-NRI (2001-35304-09991), DOE (DEFG0201ER15170) and the Plant Sciences Institute at Iowa State University.

### References

1. Brown MPS, Grundy WN, Lin D, *et al.* 2000. Knowledge-based analysis of microarray gene expression data by using support vector machines. *Proc Nat Acad Sci USA* **97**: 262–267.
2. Dickerson JA, Berleant D, Cox Z, Qi W, Wurtele ES. 2001. Creating metabolic network models using text mining and expert knowledge. Atlantic Symposium on Molecular Biology and Genome Information Systems and Technology (CBGIST 2001): 26–30.
3. Dickerson JA, Cox Z, Wurtele ES, Fulmer AW. 2001. Creating metabolic and regulatory network models using fuzzy cognitive maps. North American Fuzzy Information Processing Conference (NAFIPS) **4**: 2171–2176.
4. Dickerson JA, Kosko B. 1994. Virtual worlds as fuzzy cognitive maps. *Presence* **3**: 173–189.
5. Dickerson JA, Berleant D, Cox Z, *et al.* 2003. Creating and modelling metabolic and regulatory networks using text mining and expert knowledge. In *Computational Biology and Genome Informatics*, Wang PP, Wu CH, Wang JTL (eds). World Scientific (in press).
6. Ding J, Berleant D, Nettleton D, Wurtele E. 2002. Mining Medline: abstracts, sentences, or phrases? Pacific Symposium on Biocomputing (PSB 2002): **3–7**: 326–337.
7. Dougherty ER, Barrera J, Brun M, *et al.* 2002. Inference from clustering with application to gene-expression microarrays. *J Comput Biol* **9**: 105–126.
8. Fatland BF, Ke J, Anderson M, *et al.* 2002. Molecular characterization of a novel heteromeric ATP-citrate lyase that generates cytosolic acetyl-CoA in *Arabidopsis*. *Plant Physiol* **130**: 740–756.
9. Kanehisa M, Goto S. 2000. KEGG: Kyoto encyclopedia of genes and genomes. *Nucleic Acids Res* **28**: 27–30.
10. Karp PD, Riley M, Paley SM, Pellegrini-Toole A. 2002. The MetaCyc database. *Nucleic Acids Res* **30**: 59–61.
11. Karp PD, Riley M, Saier M, *et al.* 2002. The EcoCyc database. *Nucleic Acids Res* **30**: 56–58.
12. Oliver DJ, Nikolau BJ, Wurtele ES. Functional genomics: high throughput mRNA, protein, and metabolite analyses. *Metab Eng* **4**: 98–106.
13. Overbeek R, Larsen N, Pusch GD, *et al.* 2000. WIT: integrated system for high-throughput genome sequence analysis and metabolic reconstruction. *Nucleic Acids Res* **28**: 123–125.
14. Swayne D, Temple Lang D, Buja A, Cook D. 2003. GGobi: evolving from Xgobi into an extensible framework for interactive data visualization. *J Comp Stat Data Anal* (in press).
15. Tomita M. Whole-cell simulation: a grand challenge of the 21st century. 2001. *Trends Biotechnol* **19**: 205–210.
16. Unwin A, Hofmann H, Theus M, Siegl B, Hawkins G. 1996. MANET: missings are now equally treated. **http://www1.math.uni-augsburg.de/Manet/**
17. Yao T. 2002. Bioinformatics for the genomic sciences and towards systems biology. Japanese activities in the post-genome era. *Prog Biophys Mol Biol* **80**: 23–42.

Copyright © 2003 John Wiley & Sons, Ltd. 
